# Effects of Acupuncture on Lowering Blood Pressure in Postmenopausal Women with Prehypertension or Stage 1 Hypertension: A Propensity Score-Matched Analysis

**DOI:** 10.3390/jcm10071426

**Published:** 2021-04-01

**Authors:** Bok-Nam Seo, Ojin Kwon, Siwoo Lee, Ho-Seok Kim, Kyung-Won Kang, In Chan Seol, Chol Shin, Sun-Mi Choi

**Affiliations:** 1 Clinical Medicine Division, Korea Institute of Oriental Medicine, Daejeon 34054, Korea; florence@kiom.re.kr (B.-N.S.); cheda1334@kiom.re.kr (O.K.); hodol9980@kiom.re.kr (H.-S.K.); 2 Future Medicine Division, Korea Institute of Oriental Medicine, Daejeon 34054, Korea; bfree@kiom.re.kr; 3 Bestian Clinical Trial Center, Bestianwoosong Hospital, Daejeon 34617, Korea; gyungwon.gang@gmail.com; 4 Department of Internal Medicine, Daejeon Korean Medicine Hospital of Daejeon University, Daejeon 35235, Korea; seolinch@dju.kr; 5 Division of Pulmonary Sleep and Critical Care Medicine, Department of Internal Medicine, Korea University Ansan Hospital, Ansan 15355, Korea; chol-shin@korea.ac.kr; 6 Department of Research, Korea Institute of Oriental Medicine, 1672 Yuseongdaero, Yuseong-gu, Daejeon 34054, Korea; 7 Korean Convergence Medicine, University of Science & Technology, Daejeon 34113, Korea

**Keywords:** acupuncture, cardiovascular disease, hypertension, postmenopausal, prehypertension, propensity score-matching

## Abstract

Postmenopausal women have a higher prevalence of hypertension compared to premenopausal women. Hypertension is a risk factor for cardiovascular diseases, the prevalence of which is ever increasing. This study investigated the effects of long-term acupuncture on lowering the blood pressure of postmenopausal women with prehypertension and stage 1 hypertension. Participants were 122 postmenopausal women aged less than 65 years, diagnosed with prehypertension or stage 1 hypertension (systolic blood pressure 120–159 mmHg or diastolic blood pressure 80–99 mmHg). We used a propensity score-matched design. The experimental group (*n* = 61) received acupuncture for four weeks every six months over a period of two years. The control group (*n* = 61) received no intervention. An Analysis of covariance (ANCOVA) was performed for the primary efficacy analysis. Relative risk ratios were used to compare group differences in treatment effects. Acupuncture significantly reduced the participants’ diastolic blood pressure (−9.92 mmHg; *p* < 0.001) and systolic blood pressure (−10.34 mmHg; *p* < 0.001) from baseline to follow-up. The results indicate that acupuncture alleviates hypertension in postmenopausal women, reducing their risk of developing cardiovascular diseases and improving their health and quality of life.

## 1. Introduction

The prevalence of hypertension is higher among postmenopausal women than among both premenopausal women and men [1,2,3,4,5]. Hypertension is a major risk factor for developing cardiovascular diseases. Declining estrogen levels in postmenopausal women increase the levels of blood lipids, inducing atherosclerosis and increasing their risk of developing cardiovascular diseases [6,7,8]. The prevalence of cardiovascular disease is also higher among women than men and is specifically higher among postmenopausal women than premenopausal women [9,10]. Owing to their unique physiological characteristics, postmenopausal women are at increased risk of prehypertension and mild hypertension, therefore making lifestyle management a necessity [11].

High blood pressure (BP) guidelines from the Seventh Report of the Joint National Committee on Prevention (JNC 7) recommend managing both high BP and its associated risk factors. Prehypertension is more likely than normal BP to progress to hypertension [12,13]. Furthermore, lifestyle improvements and non-pharmacological complementary and alternative medicinal approaches, such as acupuncture, can prevent hypertension [14].

Several studies have confirmed the effect of acupuncture interventions on BP control [14,15,16,17,18] and acupuncture was found to lower BP and relieve hypertension symptoms in various cases. Therefore, acupuncture treatment may also be effective in the management of BP in postmenopausal women. However, no current empirical research has identified whether acupuncture is useful in the long-term management of hypertension.

The purpose of this study was to investigate the effect of a long-term acupuncture intervention on lowering the BP of postmenopausal women at risk of developing hypertension, namely postmenopausal women with prehypertension (systolic BP [SBP] 120–139 mmHg or diastolic BP [DBP] 80–89 mmHg) and stage 1 hypertension (SBP 140–159 mmHg or DBP 90–99 mmHg). The measured outcomes included changes in the participants’ BP levels following the acupuncture treatment.

## 2. Materials and Methods

### 2.1. Study Design and Participants

The data for this study were obtained from both the Hypertension Intervention Cohort Study (HICS) [19] and the Korea Constitution Multicenter Study (KCMS) [20] on hypertension risk. The HICS is an interventional cohort study that evaluated the effects of long-term acupuncture to prevent hypertension in postmenopausal women [19]. The KCMS is a cross-sectional study conducted by Sasang Constitutional Research in cooperation with the Korean Genome and Epidemiology Study (KoGES)—the Ansan and Ansung cohort [20,21,22].

Both studies were conducted from 2014 to 2016. Participants were 122 postmenopausal women aged less than 65 years with either established prehypertension or stage 1 hypertension. The participants were divided into experimental (*n* = 61) and control (*n* = 61) groups. The experimental group received the acupuncture treatment, while those in the control group self-managed their BP without acupuncture treatment. Both groups were observed for changes in their BP levels over two years. The control group followed their usual care-managed lifestyle habits and received no treatment. Both groups managed their own lifestyle habits related to BP.

The acupuncture group was comprised of 140 participants from the HICS. In the HICS study, 140 treatment groups were enrolled using the selection exclusion criteria, of which 109 patients completed all the acupuncture interventions within two years. In the KMCS cohort, 101 control groups were selected according to the same selection exclusion criteria as in the HICS cohort, and the two groups were designed to be identical through PSM (Propensity Score Matching). The inclusion and exclusion criteria were chosen according to the same factors in both studies.

**Figure 1:**
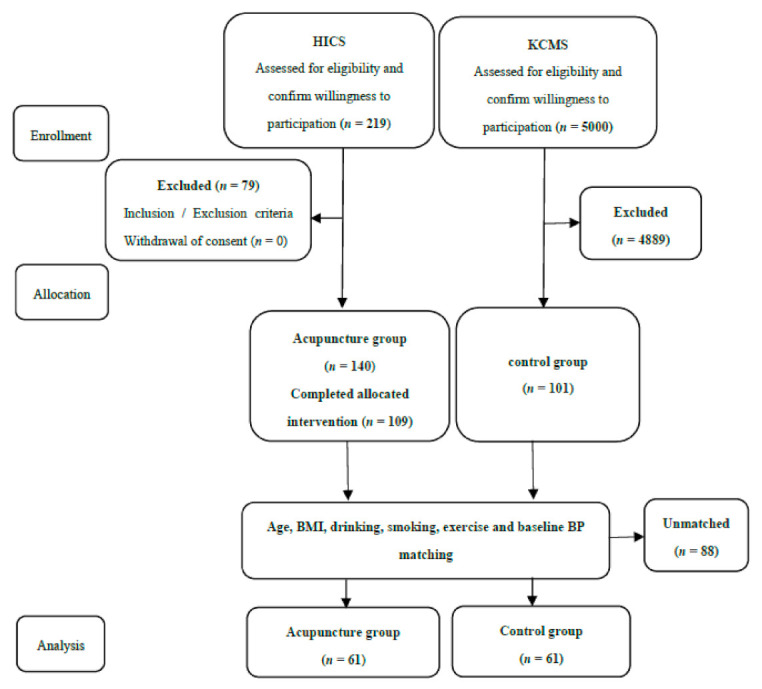
Study flow chart. HICS: Hypertension Intervention Cohort Study; KCMS: Korea Constitution Multicenter Study.

In this study, 1:1 matching was performed using a propensity score to reduce the selection bias as much as possible. The control variables used were age, body mass index (BMI), drinking, smoking, exercise and baseline BP, and the matching method used was Nearest Neighbor matching (Figure 1). Participants for whom more than one year had passed since menopause were selected according to the selection criteria. As a result, 61 participants were selected for each group; their demographic characteristics are shown in Table 1.

All participants were recruited by doctors practicing Korean medicine (KM). The details of the HICS (SPRINT) are given in the study protocol [19]. For the HICS study, we referred to Liu et al. [14]. and considered a long follow-up period (two years) and a four-session acupuncture period. The inclusion criteria included: (1) being a postmenopausal woman aged less than 65 years, (2) having prehypertension or stage 1 hypertension BP (i.e., SBP 120–159 mmHg or DBP 80–99 mmHg according to at least three manual measurements) and (3) taking no hypertension medication. The exclusion criteria were as follows: (1) having secondary hypertension (e.g., renal diseases including chronic renal failure and renal artery stenosis; or endocrine diseases including adrenal disease and thyroid disease), (2) having other biological causes of hypertension (e.g., neurological disorders, obstructive sleep apnea, cancer, infectious diseases or the use of medications that affect BP), (3) receiving ongoing treatment for cerebrovascular or heart diseases, (4) having uncontrolled diabetes mellitus, (5) having received prior hormonal therapy within the past month, (6) having received KM treatment within the past month, and (7) having had previous hypersensitive reactions to acupuncture treatment.

### 2.2. Trial Procedures

The acupuncture group received 10 acupuncture treatments over a period of four weeks with two or three treatments per week. These interventions were conducted every six months over a period of two years. The control group received no acupuncture interventions. All participants were followed from the baseline for 24 months.

Acupuncture was applied along eight acupoints: the bilateral Fengchi (GB20), the Quchi (LI11), the Zusanli (ST36) and the Sameumgyo (SP6) points. These acupoints were chosen based on a consensus among five acupuncture experts specializing in KM. These points have been used previously in two studies conducted by this research group [14,19].

The KM doctors manually manipulated the acupuncture needles to obtain de qi sensations and maintained the needles in this position for 30 min, with intermittent manual stimulation and a needle insertion depth of 3.15 mm. All the acupuncturists were licensed KM doctors with at least three years of acupuncture experience. We used sterile, disposable needles with a length of 30 mm and a diameter of 0.25 mm (Dongbang Acupuncture Inc., Poryong, Korea). BP was assessed after every treatment session.

### 2.3. Primary and Secondary Outcomes

The primary outcome of this study was the change in participants’ SBP and DBP between the baseline and the following 24 months. After 4 months of follow-up after the last acupuncture treatment, blood pressure was analyzed from the last measured blood pressure. The control group was also analyzed by measuring blood pressure at the same time point.

BP was measured using a conventional mercury sphygmomanometer (Baumanometer, USA standard type) with subjects in the sitting position and Blood pressure was measured by a clinical researcher. In the control group, BP was measured using the same methods as in the treatment group. At each BP assessment point, the patients were asked to rest for at least five minutes before undergoing their first BP measurement. Their BP was then measured again twice, at two-minute intervals [3]. For each of the treatment and the control groups, the average value of three measurements was used for the analysis. Blood pressure measurements were usually taken during morning or afternoon activity. Participants were advised to avoid caffeine-containing beverages and smoking, exercising for 30 min prior to the BP measurement and avoiding heavy food intake one day before their visit. The BP assessment was conducted using a standardized protocol for this study.

The secondary outcomes were the treatment effects, that is, acupuncture improving stage 1 hypertension to either prehypertension or normal BP and prehypertension to normal BP. We analyzed the differences in the treatment effect between the two groups and between subgroups as a relative risk ratio.

### 2.4. Statistical Analysis

All statistical analyses were performed using SAS Version 9.4 (SAS Institute Inc., Cary, NC, USA), with a significance level of *p* = 0.05 and two-sided tests; for this analysis, a total of at least 122 patients were required in the two groups. Missing data were addressed using multiple imputation. Full dataset and per-protocol analyses were conducted. We analyzed the efficacy and safety of the intervention using the intention-to-treat principle. Demographic characteristics between the two groups were compared using a two-sample independent *t*-test and Fisher’s exact test. For the primary efficacy analysis, an analysis of covariance (ANCOVA) was used, with the baseline BP as covariates and group placement as the fixed factor. To confirm that the sampling was not biased, the population was compared by multiple regression analysis. Potential confounding variables were controlled by matching the covariates of the two groups as much as possible according to the propensity scores calculated, using logistic multiple regression analysis after applying PSM (propensity scores matching). In addition, relative risk ratios were used to analyze the differences in treatment effects between the two groups, as well as between the subgroups.

### 2.5. Ethical Considerations

The study protocol was approved by the Dunsan Korean Medicine Hospital of Daejeon University, Daejeon, Korea, and the Human Subjects Review Committee at the Korea University Ansan Hospital, Ansan, Korea. All participants provided their written informed consent prior to the study. Participants were informed that their participation in the KCMS study was entirely voluntary and that refusal to participate would not affect their medical care.

## 3. Results

### 3.1. Patient Baseline Characteristics

From 2014 to 2016, patients were enrolled based on eligibility and subsequently finished their treatment and follow-up processes. A total of 122 patients were thus included in the final analysis (Figure 1). The demographic and clinical characteristics of the two groups were similar at the baseline (Table 1).

Table 1 shows the baseline characteristics of the two groups. The two groups were well balanced by propensity score matching and 122 patients were matched (Table 1). The mean age of the acupuncture group was 57.44 years, (95% confidence interval (CI) [56.51, 58.37]) and that of the control group was 57.87 years (95% CI [56.76, 58.98]). Participants in the acupuncture group did more exercise. Smoking and drinking were similar in the two groups. The diagnosis of hypertension was 11.48% of the acupuncture group, which was higher than that of the control group, and only one participants was diagnosed with hypertension but was not receiving treatment. The mean BMI was similar between the two groups (Table 1).

### 3.2. End Points

In the acupuncture group, the changes in DBP in DBP (−9.92 mmHg; *p* < 0.001) and SBP (−10.34 mmHg; *p* < 0.001) between the baseline and post-treatment were significant, and there was a significant difference in BP between the two groups at post-treatment. The mean difference was DBP (−6.44 mmHg; *p* = 0.008) and SBP (−7.53 mmHg; *p* < 0.001) (Table 2).

Multiple regression analysis (MRA) was performed to check for unbiased sampling of the population. As a result, the mean difference was DBP (−7.55 mmHg; *p* < 0.001) and SBP (−6.89 mmHg; *p* < 0.001) (Table 3). It was found to be similar to the results of ANCOVA after PSM. The sample is an unbiased estimate of the population. In the ANCOVA, SBP and DBP were adjusted for analysis (Table 2).

There were no significant differences between the two groups in the BMI scores (Table 4). Between-group differences in changes in BP from baseline to 24 months for the subgroups are shown in Table 3. The BMI relative risk ratios indicated an acupuncture effect 4.04 (95% CI [1.72, 9.52]) times higher for BMI of 25 and above. The Exercise relative risk ratios indicated an acupuncture effect 1.83 (95% CI [1.10, 3.06]) times higher for those who exercised than those who did not (Table 4).

**Figure 2:**
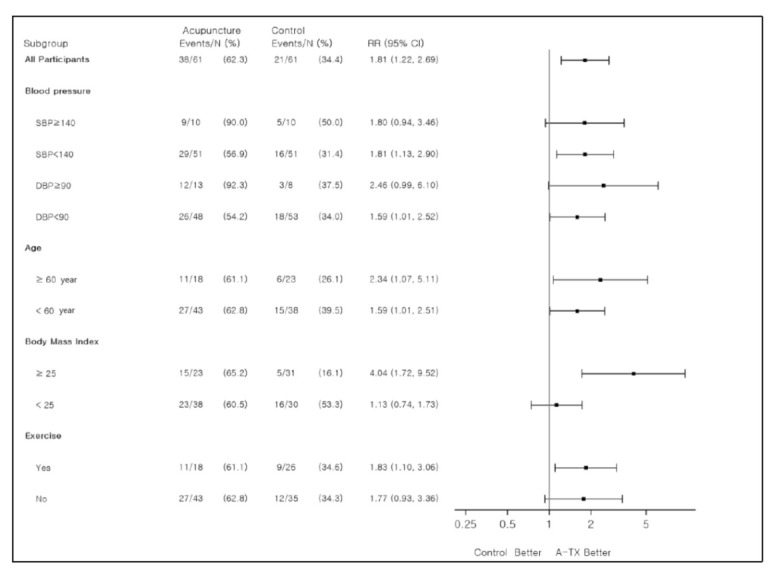
Comparison of treatment effects between the subgroups. RR: risk ratio; CI: confidence interval; SBP: systolic blood pressure; DBP: diastolic blood pressure; A-TX, acupuncture treatment.

The efficacy of long-term acupuncture treatment for postmenopausal women with hypertension was as follows: for those with DBP ≥ 90 mmHg, the relative risk was 2.46 (95% CI [0.99, 6.10]), which was the highest. The relative risk ratios for BP across the various subgroups were lower in the acupuncture group, indicating that the acupuncture group showed a greater treatment effect than the control group (Figure 2).

### 3.3. Adverse Events

No localized complications, such as bleeding, hematoma, perforation, infection, or other adverse events related to acupuncture, were observed. The most frequent complaint about acupuncture treatment was the time the visits required. Moreover, two participants dropped out of the study owing to discomfort with the acupuncture stimulus.

## 4. Discussion

Increased BP after menopause is associated with a decrease in estrogen. When this hormone decreases after menopause, the arteries do not dilate completely which causes the blood vessels to constrict and increase the BP. Therefore, menopausal women need BP management [23].

In the treatment of hypertension, problems related to long-term drug use and the side effects of drugs have been continuously raised in recent times, and interest in non-drug treatment as an alternative is increasing [24]. According to the literature, recent treatments of high blood pressure in postmenopausal women can be broadly classified into lifestyle-improvement practices such as a low-salt diet, exercise, weight reduction and drug therapy [25]. Lifestyle interventions, however, are difficult to achieve and even more difficult to maintain, and drug therapy is costly, fraught with compliance problems, and accompanied by unwanted side effects. Therefore, it is necessary to prevent hypertension and cardiovascular disease by developing non-drug therapy interventions that compensate for the disadvantages of drug therapy.

The primary advantage of acupuncture is that there is no need for long-term medication for high BP, and the therapeutic effect can be maintained even after discontinuing the medicine. It can also prevent the side effects of high BP medications. Several experiments have suggested that acupuncture reduces BP more than RAS inhibitors [26]. This study aimed to investigate the effects of long-term acupuncture as an intervention to lower BP in postmenopausal women with either prehypertension or stage 1 hypertension. The changes in SBP and DBP were analyzed in the long-term acupuncture group and the usual care control group.

Several studies using acupuncture to treat hypertension used short treatment periods of 4 to 6 weeks [14,18,27,28,29]. Our study demonstrated the effects of long-term acupuncture on BP in postmenopausal women with prehypertension or stage 1 hypertension.

Li et al. [30] reported a decrease of 6.0 mmHg in SBP and 4.0 mmHg in DBP. Liu et al. [14] reported reductions of 6.0 mmHg and 5.7 mmHg in SBP and DBP, respectively. Further, Chen et al. [18] documented reductions of 10.4 mmHg and 5.7 mmHg in SBP and DBP, respectively. In this study, SBP levels were reduced by 11.6 mmHg, and DBP by 10.7 mmHg. Therefore, long-term acupuncture treatment appears to be more effective in lowering BP than short-term acupuncture.

Acupuncture treatment and follow-up over a period of two years reduced BP in postmenopausal women, suggesting that the development of prehypertension into hypertension could be prevented by managing the condition in its first stage.

In addition, in our study, obese hypertensive participants with a BMI of 25 and above showed a greater BP reduction after undergoing the acupuncture intervention. A meta-analysis by Nestle et al. [31] revealed that a decrease of 3–9% in body weight reduced SBP by 3 mmHg and DBP by 3 mmHg. Racette et al. [32] observed that a loss of weight in overweight people lowered their BP. The incidence of hypertension was lower in the weight-loss group than in the control group [33]. Previous research has shown that losing weight reduces the risk of high BP. Our findings suggest that acupuncture is a useful intervention for lowering BP in obese postmenopausal women.

Furthermore, previous studies have shown that exercise reduces BP [34,35]. However, in our study, there was no difference in the effectiveness of acupuncture with exercise. A limitation of our study was that exercise was not controlled.

This study has several strengths. First, we analyzed the effects of acupuncture treatment in preventing hypertension in postmenopausal women over a long period (two years). Hypertension is a major risk factor for cardiovascular disease and occurs more in postmenopausal women than in premenopausal women. In postmenopausal women, long-term acupuncture treatment is a useful treatment for high blood pressure and is effective in alleviating the prevalence of high blood pressure.

Second, the acupuncture procedure in this study was used as an alternative to medication, along with lifestyle management strategies, as a method for preventing the development of hypertension. Third, Acupuncture improved the control of BP levels in postmenopausal women, reducing their risk of developing cardiovascular diseases.

### Limitation

There are several limitations in this study. First, we matched using Propensity Score Matching (PSM) in HICS and KCMS studies to control any confusion of the data. Nevertheless, the two groups cannot be completely matched due to unknown influencing factors.

Second, the control group was not designed with the same regular session as the treatment group. The control group only measured blood pressure at the beginning and end. Participants in the clinical trial as a control group can expect to see a blood pressure lowering effect without acupuncture treatment. Future studies suggest supplementing these points to the study design.

Finally, the participants could not be followed up after the study ended. However, the findings confirming the prevention of hypertension in the participants over two years after the onset of menopause indicate that acupuncture contributes to lowering the risk of future cardiovascular diseases in postmenopausal women, thus improving their overall health condition and quality of life. Hence, acupuncture is suggested as one of the alternative treatments for BP control in postmenopausal women. Further long-term follow-up studies are needed to confirm these findings.

## 5. Conclusions

This study offers evidence that acupuncture treatment might have beneficial effects in reducing the risk of hypertension in postmenopausal women and, by lowering BP, reduce the risk of developing cardiovascular diseases.

## Figures and Tables

**Table 1:** Participants’ baseline characteristics.

Characteristic	Acupuncture Group (*n* = 61)	Control Group (*n* = 61)	*p*
Age (years)	57.44 (56.51, 58.37)	57.87 (56.76, 58.98)	0.557 ^a^
≥60 years	18 (29.51%)	23 (37.70%)	0.444 ^b^
<60 years	43 (70.49%)	38 (62.30%)	
BMI (kg/m^2^)	24.48 (23.74, 25.21)	25.05 (24.26, 25.83)	0.292 ^a^
≥25	23 (37.70%)	31 (50.82%)	0.202 ^b^
<25	38 (62.30%)	30 (49.18%)	
Vital signs			
SBP (mmHg)	130.2 (127.7, 132.8)	128.4 (125.6, 131.1)	0.321 ^a^
≥140	10 (16.39%)	10 (16.39%)	0.999 ^b^
<140	51 (83.61%)	51 (83.61%)	
DBP (mmHg)	84.57 (82.94, 86.20)	83.94 (82.11. 85.17)	0.405 ^a^
≥90	13 (21.31%)	8 (13.11%)	0.338 ^b^
< 90	48 (78.69%)	53 (86.89%)	
Smokes (Y/N)	1 (1.64%)/60 (98.36%)	1 (1.64%)/60 (98.36%)	0.999 ^b^
Drinks (Y/N)	14 (22.95%)/47 (77.05%)	17 (27.9%)/44 (72.13%)	0.678 ^b^
Exercises (Y/N)	43 (70.49%)/18 (29.51%)	35 (57.38%)/26 (42.62%)	0.187 ^b^
Medical history			
Hypertension (Y/N)	7 (11.48%)/54 (88.52%)	4 (6.56%)/57 (93.44%)	0.529 ^b^
Diabetes (Y/N)	0 (0.00%)/61 (100.0%)	4 (6.56%)/57 (93.44%)	0.119 ^b^
Hyperlipidemia (Y/N)	10 (16.39%)/51 (83.61%)	12 (19.67%)/49 (80.33%)	0.814 ^b^
Surgery (Y/N)	24 (39.34%)/37 (60.66%)	28 (45.90%)/33 (54.10%)	0.583 ^b^

Abbreviation: BMI, body mass index; TG, triglyceride; HDL, high-density lipoprotein; SBP, systolic blood pressure; DBP, diastolic blood pressure; Y, yes; N, no. Data are means (95% confidence interval) for continuous variables and frequencies (percentages) for categorical variables; a = Student’s independent *t*-test; b = Fisher’s exact test.

**Table 2:** Comparison of blood pressure changes between and within groups.

Vital Signs	Acupuncture Group (*n* = 61)	Control Group (*n* = 61)	Mean Difference ^a^	*p ^b^*
SBP				
Baseline	130.2 (127.7, 132.8)	128.4 (125.6, 131.1)		
After 2 years	119.9 (117.1, 122.7)	125.4 (121.2, 129.6)	−6.44 (−11.17, −1.71)	0.008 **
Difference	−10.34 (−13.55, −7.14)	−2.92 (−6.93, 1.10)		
*p* ^c^	<0.001 ***	0.151		
DBP				
Baseline	84.6 (82.9, 86.2)	83.9 (82.1. 85.2)		
After 2 years	74.6 (72.7, 76.6)	81.5 (78.6, 84.3)	−7.53 (−10.52, −4.55)	<0.001 ***
Difference	−9.92 (−11.89, −7.96)	−2.16 (−4.49, 0.16)		
*p* ^c^	<0.001 ***	0.068		

Abbreviation: SBP, systolic blood pressure; DBP, diastolic blood pressure. a = Least squares mean difference using analysis of covariance (ANCOVA); b = *p*-value from ANCOVA; c = *p*-value from paired *t*-test; ** *p* < 0.01; and *** *p* < 0.001.

**Table 3:** Comparison of blood pressure changes between and within groups (Population).

Vital Signs	Acupuncture Group (*n* = 109)	Control Group (*n* = 101)	Mean Difference ^a^	*p* ^b^
SBP				
Baseline	133.2 (131.2, 135.1)	128.9 (126.6, 131.2)		
After 2 years	121.3 (118.9, 123.7)	126.91 (123.8, 130.1)	−7.55 (−11.35, −3.75)	<0.001 ***
Difference	−11.92 (−14.50, −9.35)	−1.94 (−4.96, 1.08)		
*p* ^c^	<0.001 ***	0.206		
DBP				
Baseline	87.5 (86.3, 88.8)	82.9 (81.5, 84.4)		
After 2 years	76.6 (74.9, 78.3)	80.6 (78.5, 82.6)	−6.89 (−9.26, −4.52)	<0.001 ***
Difference	−10.89 (−12.47, −9.31)	−2.36 (−4.05, −0.66)		
*p* ^c^	<0.001 ***	0.007 **		

Abbreviation: SBP, systolic blood pressure; DBP, diastolic blood pressure. a = mean difference using Multiple regression analysis (control variables: age, BMI, baseline BP, Drinks, smokes, Exercise); b = *p*-value from Multiple regression analysis; c = *p*-value from paired *t*-test; **: *p* < 0.01; and ***: *p* < 0.001.

**Table 4:** Comparison of changes between groups according to blood pressure level and other characteristics.

Subgroup	Acupuncture Group	Control Group	Relative Risk Ratio
All participants	62.3% (49.8%, 73.4%)	34.4% (23.8%, 47.0%)	1.81 (1.22, 2.69)
Blood pressure			
SBP ≥ 140	90.0% (59.6%, 98.2%)	50.0% (23.7%, 76.3%)	1.80 (0.94, 3.46)
SBP < 140	56.9% (43.3%, 69.5%)	31.4% (20.3%, 45.0%)	1.81 (1.13, 2.90)
DBP ≥ 90	92.3% (66.7%, 98.6%)	37.5% (13.7%, 69.4%)	2.46 (0.99, 6.10)
DBP < 90	54.2% (40.3%, 67.4%)	34.0% (22.7%, 47.4%)	1.59 (1.01, 2.52)
Age (years)			
≥60	61.1% (38.6%, 79.7%)	26.1% (12.6%, 46.5%)	2.34 (1.07, 5.11)
55–59	53.6% (35.8%, 70.5%)	33.3% (17.2%, 54.6%)	1.61 (0.80, 3.22)
<55	80.0% (54.8%, 93.0%)	47.1% (26.2%, 69.0%)	1.70 (0.97, 2.99)
Body mass index			
≥25	65.2% (44.9%, 81.2%)	16.1% (7.1%, 32.6%)	4.04 (1.72, 9.52)
<25	60.5% (44.7%, 74.4%)	53.5% (36.1%, 69.8%)	1.13 (0.74, 1.73)
Exercise			
Yes	61.9% (46.8%, 75.0%)	37.5% (13.7%, 69.4%)	1.65 (0.65, 4.17)
No	63.2% (41.0%, 80.9%)	34.0% (22.7%, 47.4%)	1.86 (1.12, 3.09)

Abbreviation: SBP, systolic blood pressure; DBP, diastolic blood pressure. Data are the estimated proportion (95% confidence interval for proportion) of participants showing a reduction in BP for each group. Event means that stage 1 hypertension was improved to pre-hypertension or to normal BP, and pre-hypertension to normal BP.

## Abbreviations

BMI	body mass index
BP	blood pressure
CI	confidence interval
DBP	diastolic blood pressure
HICS	Hypertension Intervention Cohort Study
KM	Korean medicine
KoGES	Korea Genome and Epidemiology Study
KCMS	Korea Constitution Multicenter Study
SBP	systolic blood pressure
